# Isolation, Identification and In Silico Evaluation of Novel Cholinesterase Inhibitors from *Terminalia triptera* Stapf.

**DOI:** 10.3390/molecules31071113

**Published:** 2026-03-27

**Authors:** Tu Quy Phan, Hung Tse Huang, San-Lang Wang, Dinh Sy Nguyen, Manh Dung Doan, Thi Huyen Thoa Pham, Thi Kim Thu Phan, Ba Phong Truong, Van Bon Nguyen

**Affiliations:** 1 Department of Science and Technology, Tay Nguyen University, Dak Lak 630000, Vietnam; phantuquy@ttn.edu.vn (T.Q.P.); ndsy@ttn.edu.vn (D.S.N.); pththoa@ttn.edu.vn (T.H.T.P.); ptkthu@ttn.edu.vn (T.K.T.P.); tbphong@ttn.edu.vn (B.P.T.); 2 Division of Chinese Materia Medica Development, National Research Institute of Chinese Medicine, Taipei 11221, Taiwan; kk49310953@nricm.edu.tw; 3 Department of Chemistry, Tamkang University, New Taipei City 25137, Taiwan; 4 Life Science Development Center, Tamkang University, New Taipei City 25137, Taiwan; 5 Institute of Biotechnology and Environment, Tay Nguyen University, Dak Lak 630000, Vietnam; dmdung@ttn.edu.vn

**Keywords:** *Terminalia triptera*, cholinesterase inhibitors, Alzheimer’s disease, DFT, molecular docking, molecular dynamics

## Abstract

Alzheimer’s disease (AD) remains a significant global health challenge, highlighting the need for novel dual inhibitors targeting acetylcholinesterase (AChE) and butyrylcholinesterase (BChE). This study investigated the trunk bark of *Terminalia triptera* Stapf. as a potential source of bioactive secondary metabolites for AD management. Bioassay-guided isolation led to the identification of two flavan-3-ol derivatives, epicatechin-(4β→8)-*ent*-catechin (**1**) and (−)-catechin (**2**), reported here for the first time from this species. In vitro assays demonstrated that the dimeric compound **1** exhibited stronger dual inhibitory activity against AChE and BChE, with IC_50_ values of 4.41 × 10^−4^ and 4.75 × 10^−4^ mol/L, respectively, surpassing the reference compound berberine chloride. Molecular docking analysis revealed that compound **1** formed extensive interactions within both catalytic and peripheral anionic sites of the enzymes. Density Functional Theory (DFT) calculations indicated high kinetic stability, reflected by large HOMO–LUMO energy gaps (6.66–6.97 eV), while global reactivity descriptors suggested lower electrophilicity (ω = 2.19–2.34 eV), supporting a potentially favorable safety profile. Furthermore, 100 ns molecular dynamics simulations confirmed stable ligand–protein complexes stabilized by hydrogen-bond networks and deep binding within catalytic pockets. Overall, these findings highlight *T. triptera* and its dimeric proanthocyanidins as promising multi-target candidates for anti-Alzheimer drug development.

## 1. Introduction

Alzheimer’s disease (AD) is the most prevalent neurodegenerative disorder and the primary etiology of dementia worldwide, accounting for approximately 60–70% of all cases [1]. Currently, an estimated 57 million individuals globally live with dementia, with nearly 10 million new diagnoses annually—a figure projected to escalate sharply due to the accelerating aging of the global population [1,2]. Beyond progressive cognitive decline and memory impairment, AD severely compromises activities of daily living (ADL), autonomy, and social engagement, ultimately culminating in profound disability and increased mortality. Indeed, dementia has emerged as a leading cause of disability-adjusted life years (DALYs) among older adults and is ranked among the top global causes of death, posing a critical challenge to public health systems [1]. The socioeconomic burden of AD is substantial; global costs reached approximately USD 1.3 trillion in 2019 and are anticipated to rise further [1,3]. These expenditures encompass clinical care, long-term institutional support, and informal caregiving, exerting immense pressure on healthcare infrastructures, particularly in low- and middle-income countries [1,2,3,4].

Pathologically, AD is characterized by complex, multifactorial mechanisms, including the extracellular accumulation of amyloid-beta (Aβ) plaques, intracellular hyperphosphorylation of tau proteins, oxidative stress, neuroinflammation, and progressive synaptic dysfunction [5,6,7]. Among the prevailing pathogenic frameworks, the cholinergic hypothesis remains one of the most rigorously validated, asserting that the degeneration of cholinergic neurons and the subsequent depletion of acetylcholine levels are pivotal contributors to cognitive deficits [8]. Consequently, the pharmacological inhibition of acetylcholinesterase (AChE) and butyrylcholinesterase (BChE) constitutes a cornerstone therapeutic strategy to enhance cholinergic neurotransmission and mitigate symptomatic decline [9]. Although several cholinesterase inhibitors (ChEIs), such as donepezil, rivastigmine, and galantamine, are clinically established, their efficacy remains symptomatic rather than disease-modifying. Furthermore, their associated side effects and inability to arrest disease progression underscore the imperative for the discovery of novel, safer, and more effective therapeutic agents [10].

Natural products derived from medicinal flora remain a reservoir for drug discovery, offering structurally diverse scaffolds with potent neuroprotective and cholinesterase inhibitory properties [11,12,13]. Bio-guided isolation coupled with in silico molecular docking has emerged as a robust methodology for identifying novel bioactive secondary metabolites and elucidating their molecular interactions with therapeutic targets. In this context, the genus *Terminalia* (Combretaceae) has garnered significant interest due to its rich phytochemical diversity and broad spectrum of pharmacological activities, notably its antioxidant and neuroprotective effects [14,15]. Despite this potential, systematic investigations specifically targeting the cholinesterase inhibitory constituents of *Terminalia triptera* Stapf. remain sparse.

In recent years, in silico approaches have become indispensable tools in modern drug discovery, enabling the rapid identification and optimization of potential therapeutic candidates. Computational strategies, including both ligand-based and structure-based virtual screening, facilitate the efficient exploration of chemical space and prioritization of bioactive compounds [16,17]. Molecular docking provides initial insights into binding modes, whereas molecular dynamics (MD) simulations further evaluate the stability and dynamic behavior of protein–ligand complexes under near-physiological conditions [18]. In addition, ADMET predictions enable early assessment of pharmacokinetic and safety profiles, thereby improving the efficiency of lead selection [19].

In this context, the present study integrates bioassay-guided isolation with computational approaches to investigate cholinesterase inhibitors from *Terminalia triptera*. This combined strategy aims to identify structurally characterized compounds with potential neuroprotective activity and to provide a scientific basis for their further development as candidate agents for Alzheimer’s disease management.

## 2. Results and Discussion

### 2.1. New Records of Terminalia triptera Extracts as Potential Sources of Cholinesterase Inhibitors

The cholinesterase inhibitory activities of *Terminalia triptera* extract were evaluated and presented in Table 1. The trunk bark extract of *T. triptera* exhibited moderate inhibitory effects against both acetylcholinesterase (AChE) and butyrylcholinesterase (BChE), with IC_50_ values of 335.42 ± 8.62 µg/mL and 405.35 ± 9.03 µg/mL, respectively. Notably, the extract demonstrated dual inhibition toward both cholinesterase enzymes, which is considered beneficial for potential anti-Alzheimer’s drug development since BChE activity increases during the later stages of Alzheimer’s disease.

Berberine chloride was used as the positive control due to its reported inhibitory activity against both AChE and BChE and its suitability for comparison with plant-derived compounds. As a natural compound with moderate potency, it provides a more appropriate benchmark than highly potent clinical inhibitors for phytochemical studies. Notably, the IC_50_ values of the *Terminalia triptera* extract were comparable to those of berberine chloride (AChE: 348.28 ± 5.78 µg/mL; BChE: 368.35 ± 6.43 µg/mL), indicating a similar level of moderate inhibitory activity within the same potency range.

Several studies have reported AChE inhibitory activities of medicinal plants collected from the Central Highlands of Vietnam, with AChE IC_50_ values ranging from approximately 76.8 to 730.6 µg/mL, depending on plant species and parts used (Table 1) [20,21,22,23]. In these reports, most extracts were primarily evaluated for AChE inhibition, while BChE activity was less frequently assessed. In this context, the present study provides additional evidence by demonstrating that *Terminalia triptera* trunk bark extract exhibits inhibitory effects against both AChE (IC_50_ = 335.42 ± 8.62 µg/mL) and BChE (IC_50_ = 405.35 ± 9.03 µg/mL). The simultaneous evaluation of both enzymes is noteworthy, as BChE plays an increasingly important role during the later stages of Alzheimer’s disease. Therefore, these findings suggest that *T. triptera* represents a promising medicinal plant from the Central Highlands with potential relevance for cholinesterase-targeted research.

The genus *Terminalia* is well known for its rich phytochemical diversity and a wide range of pharmacological activities, including neuroprotective effects [14,15]. Several species within this genus have also been reported to exhibit potential acetylcholinesterase (AChE) and butyrylcholinesterase (BChE) inhibitory activities [14,15,24,25,26]. However, to the best of our knowledge, this study is the first to report the potential cholinesterase inhibitory activity of *Terminalia triptera* Stapf., highlighting its novelty and suggesting promising prospects for further neuropharmacological investigations.

### 2.2. Isolation and Chemical Structures Elucidation of Bioactive Compounds from Terminalia triptera

**Scheme 1:**
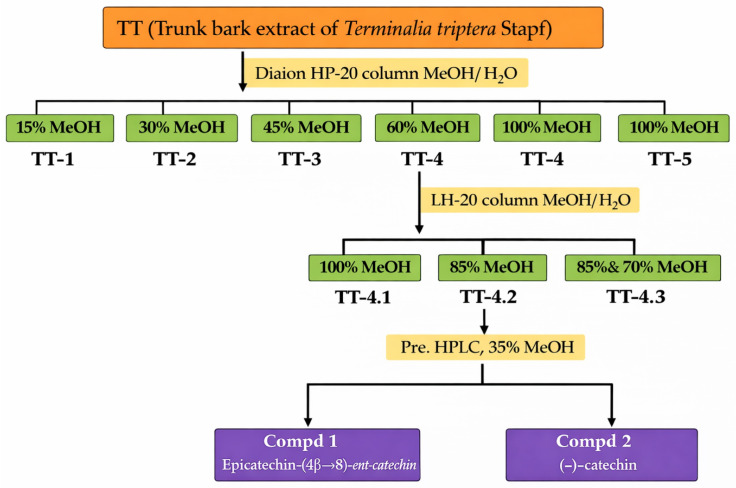
A scheme of purification and identification of bioactive inhibitors from the MeOH extract of *Terminalia triptera* (TT). The purified compounds were epicatechin-(4β→8)-*ent*-catechin (compd **1**) and (−)-catechin (compd **2**).

The bioactive constituents were isolated from the stem bark of *Terminalia triptera* through a bioassay-guided fractionation strategy targeting cholinesterase inhibitory activity (Scheme 1). As summarized in Table S1 (Supplementary Material), the crude extract (TT) exhibited moderate inhibitory effects against both AChE and BChE at the tested concentration, showing inhibition rates of approximately 81% and 78%, respectively, thereby supporting its further fractionation.

The crude extract was subsequently fractionated using Diaion HP-20 column chromatography with a MeOH/H_2_O gradient to afford five fractions (TT-1 to TT-5), all of which were evaluated under the same conditions (Table S1). Among them, TT-4 displayed the strongest activity, with inhibition rates of approximately 85% (AChE) and 81% (BChE), indicating enrichment of active constituents. Accordingly, TT-4 was selected for further purification.

Further separation of TT-4 using Sephadex LH-20 chromatography yielded three sub-fractions (TT-4.1–TT-4.3). Notably, TT-4.2 exhibited the highest inhibitory activity, with inhibition values of 97% (AChE) and 95% (BChE), suggesting that the major bioactive compounds were concentrated in this fraction. Subsequent purification of TT-4.2 by preparative HPLC led to the isolation of two pure compounds (Compounds **1** and **2**). Compound **1** showed inhibition values of 98% (AChE) and 93% (BChE), whereas Compound **2** exhibited lower inhibition values of 93% and 94%, respectively (Table S1).

The chemical structures of the isolated compounds were elucidated through comprehensive spectroscopic analysis, including nuclear magnetic resonance (NMR) and mass spectrometry (MS), followed by comparison with previously reported spectral data. Based on these analyses, the flavan-3-ol derivatives compounds were identified as epicatechin-(4β→8)-*ent*-catechin (**1**) [27] and (−)-catechin (**2**) [28].

Epicatechin-(4β→8)-*ent*-catechin (**1**) was obtained as a yellow amorphous powder; HESI-MS *m/z*: 577.1356 [M − H]^−^; ^1^H-NMR (600 MHz, pyridine-*d*_5_): δ 6.11 (1H, brs, H-2), δ 4.86 (1H, brs, H-3), δ 5.83 (1H, brs, H-4), δ 6.57 (1H, brs, H-7), δ 6.69 (1H, brs, H-9), δ 7.95 (1H, brs, H-2′), δ 7.38 (1H, brs, H-5′), δ 7.10 (1H, brs, H-6′), δ 5.18 (1H, brs, H-2″), δ 4.35 (1H, ddd, *J* = 6.0, 12.0 Hz, H-3″), δ 3.49 (1H, dd, *J* = 3.6, 16.0 Hz, H-4″a), δ 3.27 (1H, dd, *J* = 8.4, 16.0 Hz, H-4″b), δ 6.57 (1H, brs, H-7″), δ 7.95 (1H, brs, H-2‴), δ 7.38 (1H, brs, H-5‴), δ 7.10 (1H, brs, H-6‴); ^13^C-NMR (150 MHz, MeOH-*d*_4_): δ 77.5 (C-2), 72.8 (C-3), 37.4 (C-4), 102.2 (C-5), 158.3 (C-6), 96.0 (C-7), 158.5 (C-8), 95.4 (C-9), 159.0 (C-10), 132.9 (C-1′), 116.4 (C-2′), 146.4 (C-3′), 146.1 (C-4′), 115.6 (C-5′), 119.1 (C-6′), δ 82.5 (C-2″), 67.8 (C-3″), 29.1 (C-4″), 101.3 (C-5″), 155.3 (C-6″), 97.1 (C-7″), 156.3 (C-8″), 108.5 (C-9″), 154.5 (C-10″), 132.1 (C-1‴), 115.8 (C-2‴), 146.1 (C-3′), 146.0 (C-4′), 115.4 (C-5′), 119.0 (C-6′). The High-resolution ESI-MS, ^1^H NMR (600 MHz, pyridine-*d*_5_), and ^13^C NMR (150 MHz, MeOH-*d*_4_) spectra of compound **1** are shown in Figures S1–S3, respectively.

(−)-catechin (**2**) was obtained as a white amorphous powder; HESI-MS *m/z*: 289.0720 [M − H]^−^; ^1^H-NMR (500 MHz, MeOH-*d*_4_): δ 4.84 (1H, brs, H-2), δ 4.20 (1H, m, H-3), δ 2.88 (1H, dd, *J* = 4.5, 16.0 Hz, H-4a), δ 2.75 (1H, dd, *J* = 3.0, 16.0 Hz, H-4b), δ 5.96 (1H, d, *J* = 2.0 Hz, H-7), δ 5.94 (1H, d, *J* = 2.0 Hz, H-9), δ 6.70 (1H, d, *J* = 2.0 Hz, H-2′), δ 6.78 (1H, d, *J* = 8.0 Hz, H-5′), δ 6.82 (1H, ddd, *J* = 1.0, 2.0, 8.0 Hz, H-6′); ^13^C-NMR (125 MHz, MeOH-*d*_4_): δ 78.5 (C-2), 66.1 (C-3), 27.9 (C-4), 98.7 (C-5), 156.6 (C-6), 95.0 (C-7), 156.3 (C-8), 94.5 (C-9), 156.0 (C-10), 130.9 (C-1′), 113.9 (C-2′), 144.6 (C-3′), 144.4 (C-4′), 114.5 (C-5′), 118.0 (C-6′). The High-resolution ESI-MS, ^1^H NMR (500 MHz, pyridine-*d*_4_), and ^13^C NMR (125 MHz, MeOH-*d*_4_) spectra of compound **2** are shown in Figures S4–S6, respectively.

**Figure 1:**
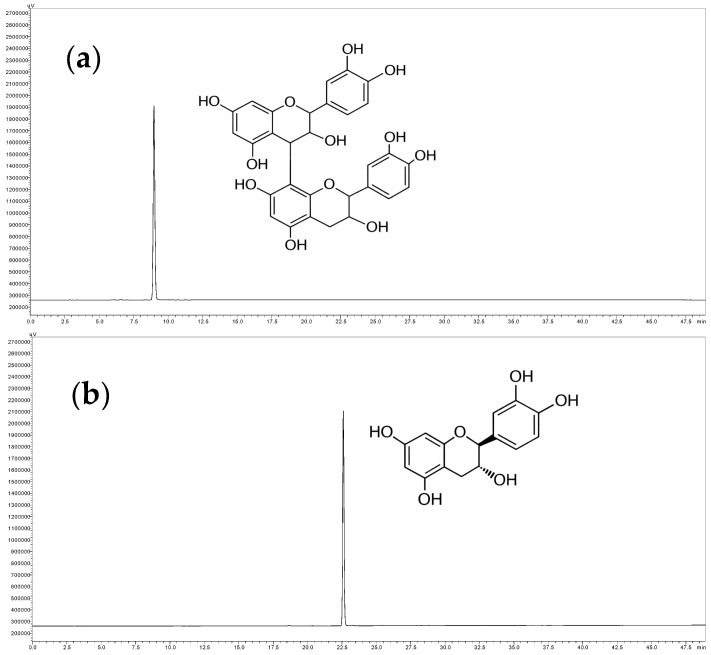
The high-performance liquid chromatography finger printings of epicatechin-(4β→8)-*ent*-catechin (**a**) and (*−*)-catechin (**b**) purified from the MeOH extract of *Terminalia triptera* Stapf.

The HPLC chromatographic profiles of the isolated compounds (Figure 1) showed well-resolved major peaks with distinct retention times (RT), supporting successful purification and high chemical homogeneity. For epicatechin-(4β→8)-*ent*-catechin (Figure 1a), the chromatogram displayed a dominant sharp peak at an RT of approximately ~9 min, characterized by a symmetrical peak shape and a stable baseline, with only negligible minor signals detected, indicating minimal co-eluting impurities. Similarly, (−)-catechin (Figure 1b) exhibited a prominent major peak at an RT of approximately ~22.5 min, suggesting efficient chromatographic separation under the applied conditions. The chromatographic results demonstrate that epicatechin-(4β→8)-ent-catechin (a) and (−)-catechin (b) were successfully purified and are suitable for subsequent biological activity evaluation and further natural product studies.

The isolation and structural elucidation of the flavan-3-ol derivatives epicatechin-(4β→8)-*ent*-catechin (**1**) and (−)-catechin (**2**) from *Terminalia triptera* represent a noteworthy addition to the phytochemical profile of this species. To the best of our knowledge, these compounds have not previously been reported as isolated constituents of *T. triptera*, as earlier studies mainly focused on extract-level bioactivity rather than detailed compound characterization. Flavan-3-ols such as catechin and epicatechin are well documented in several *Terminalia* species, including *T. chebula*, *T. arjuna*, and related taxa, where they contribute to antioxidant and pharmacological activities [15,29,30]. The occurrence of these metabolites in *T. triptera* is therefore consistent with the chemotaxonomic features of the genus, which is rich in tannins and proanthocyanidin-type polyphenols [15,31]. Nevertheless, the present work provides the first structural evidence supporting the presence of these flavan-3-ol derivatives in this species, thereby expanding its known chemical diversity and offering a plausible basis for its reported biological activities.

### 2.3. Evaluation of the Cholinesterase Inhibitory Effect of Purified Compounds from Terminalia triptera Stapf.

The cholinesterase inhibitory activities of the isolated compounds from *Terminalia triptera* were evaluated against acetylcholinesterase (AChE) and butyrylcholinesterase (BChE), and the results are summarized in Table 2. Both compounds exhibited measurable inhibitory effects toward the two enzymes, although differences in potency were observed.

(−)-Catechin (**2**) exhibited slightly lower cholinesterase inhibitory activity than the positive control, berberine chloride, with IC_50_ values of 11.9 × 10^−4^ mol/L against AChE and 12.1 × 10^−4^ mol/L against BChE, compared to 8.97 × 10^−4^ mol/L and 9.32 × 10^−4^ mol/L, respectively, for berberine chloride. These results are consistent with previous studies indicating that (−)-catechin possesses cholinesterase inhibitory activity and may contribute to the overall bioactivity of flavan-3-ol-rich extracts. Furthermore, earlier reports suggest that catechin derivatives may exhibit enhanced inhibitory effects through synergistic interactions with other flavan-3-ols [32]. Epicatechin-(4β→8)-*ent*-catechin (**1**) demonstrated inhibitory activity against both AChE and BChE, with IC_50_ values of 4.41 × 10^−4^ and 4.75 × 10^−4^ mol/L, respectively, indicating a higher potency than that of compound **2**. The improved activity of compound 1 relative to the monomeric catechin may be attributed to its dimeric structure, which could facilitate stronger interactions with the enzyme binding sites through increased molecular size and additional hydroxyl functionalities. Both compounds displayed similar inhibition toward AChE and BChE, suggesting a relatively non-selective cholinesterase inhibition profile.

The inhibitory activity of isolated compounds was observed in the high micromolar range, which is consistent with previous reports for flavan-3-ol derivatives that generally exhibit weak AChE and BChE inhibition due to their low binding affinity [32,33]. However, given the relatively high IC_50_ values, the potential contribution of non-specific effects such as colloidal aggregation cannot be excluded [34]. Therefore, the observed inhibition may reflect low-affinity on-target interactions, although further studies (e.g., detergent-based assays or DLS analysis) are required to clarify the underlying mechanism.

To the best of our knowledge, this study represents the first report of dual AChE and BChE inhibitory activity for epicatechin-(4β→8)-*ent*-catechin. These findings highlight the potential importance of flavan-3-ol dimerization in enhancing cholinesterase inhibition and provide new insights into the bioactive constituents of *Terminalia triptera*.

### 2.4. In Silico Evaluation of Cholinesterase Inhibitors Isolated from Terminalia triptera

#### 2.4.1. Molecular Docking

**Figure 2:**
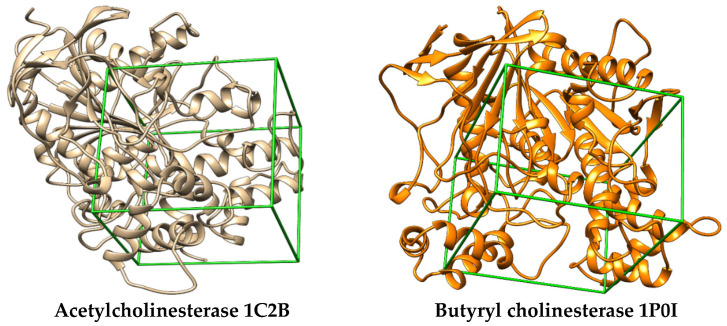
3D visualization of the docking grid box setup covering the active sites of AChE and BChE.

Molecular docking studies were conducted to evaluate the binding interactions between phytochemical constituents isolated from *Terminalia triptera* and two cholinesterase enzymes relevant to Alzheimer’s disease, namely acetylcholinesterase (AChE, PDB ID: 1C2B) and butyrylcholinesterase (BuChE, PDB ID: 1P0I). The binding pockets were carefully defined to encompass the key catalytic regions (Figure 2). For AChE, the grid box was centered at (x = 33.22, y = 84.30, z = 28.29) with dimensions of 29.96 × 30.00 × 31.77 Å. For BuChE, the grid box center was set at (x = 131.91, y = 113.09, z = 40.36) with dimensions of 40.00 × 24.40 × 31.76 Å. This configuration ensured coverage of both the Catalytic Anionic Site (CAS) and the Peripheral Anionic Site (PAS), facilitating the identification of potential dual-site inhibitors.

**Figure 3:**
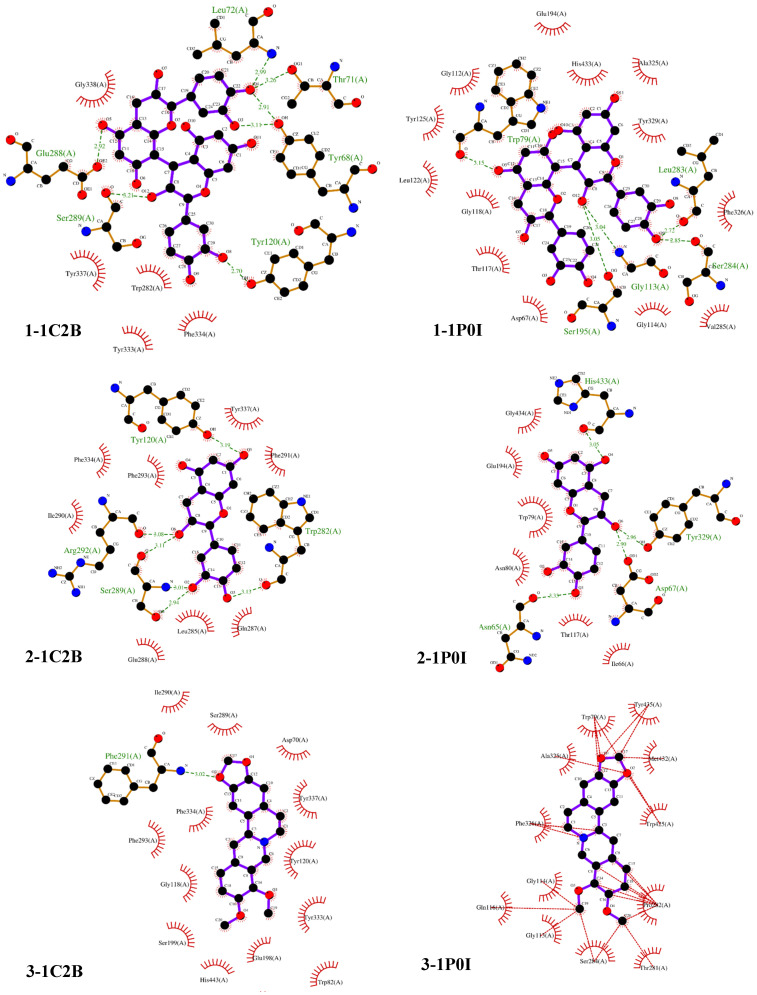
2D interaction diagrams of compounds including epicatechin-(4β→8)-*ent*-catechin (**1**), (−)-catechin (**2**), and berberine chloride (**3**) with Acetylcholinesterase (1C2B) and Butyrylcholinesterase (1P0I).

Molecular docking is widely employed as a computational screening approach to predict the binding affinity of ligands toward target enzymes, with docking scores lower than −3.20 kcal/mol generally indicating potential inhibitory activity [35]. As summarized in Table 3, all tested ligands, including epicatechin-(4β→8)-*ent*-catechin (**1**), (−)-catechin (**2**), and berberine chloride (**3**), displayed favorable binding energies against both acetylcholinesterase (AChE; −9.8 to −8.3 kcal/mol) and butyrylcholinesterase (BuChE; −11.9 to −8.7 kcal/mol). Among them, the dimeric compound epicatechin-(4β→8)-*ent*-catechin (**1**) exhibited the strongest predicted binding affinity toward both targets, with docking scores of −9.8 kcal/mol for AChE and −11.9 kcal/mol for BuChE, outperforming its monomeric counterpart (−)-catechin (**2**) (−8.3 and −9.1 kcal/mol, respectively) as well as the reference drug berberine chloride (**3**) (−8.5 and −8.7 kcal/mol). Thermodynamically, the markedly lower binding energy of compound **1**, particularly against BuChE, suggests that its bulky dimeric scaffold is structurally suited to occupy the large hydrophobic binding pocket, thereby forming a more stable ligand–protein complex [36]. These in silico results are consistent with the experimental findings presented in Table 2. Detailed ligand–enzyme interaction mechanisms are illustrated in Figure 3 and summarized in Table 3.

As illustrated in Figure 2, compound **1** adopts a conformation that blocks the entrance of the AChE gorge and establishes a strong interaction network with peripheral anionic site (PAS) residues, including Tyr120, Leu72, and Thr71. The interaction with Tyr120 is particularly important, as this residue acts as a gatekeeper controlling substrate entry [37]. By anchoring at the PAS region, compound **1** likely prevents acetylcholine from accessing the catalytic site. In contrast, the reference compound berberine chloride forms only a single major interaction with Phe291 and fails to generate an effective steric blockade, consistent with its weaker binding affinity (−8.5 kcal/mol). Compound 2 shows a distinct binding pattern, interacting mainly with peripheral and mid-gorge residues (Tyr120, Trp282, Ser289, and Arg292) through hydrogen bonds and hydrophobic contacts. However, its orientation does not form a strong steric blockade or penetrate deeply into the catalytic center, suggesting weaker inhibitory potential.

Within BChE (PDB ID: 1P0I), compound 1 exhibits a distinct binding mode compared with C3. While C3 interacts mainly with the peripheral hydrophobic pocket (Tyr435, Trp425), compound **1** penetrates deeper into the catalytic region and forms hydrogen bonds with Ser195 (a key residue of the catalytic triad) and Gly113 in the oxyanion hole [38]. This binding pattern indicates interaction with key catalytic residues. However, it should be noted that this interpretation is based solely on molecular docking and does not provide definitive evidence of the inhibition mechanism. Experimental enzymatic kinetics would be required to confirm this. In contrast, compound **2** (monomer) and berberine chloride do not show interactions with Ser195, suggesting that the dimeric structure of compound **1** may facilitate deeper access to the catalytic pocket of BChE.

#### 2.4.2. Frontier Molecular Orbitals (FMO) and Global Reactivity Descriptors

**Figure 4:**
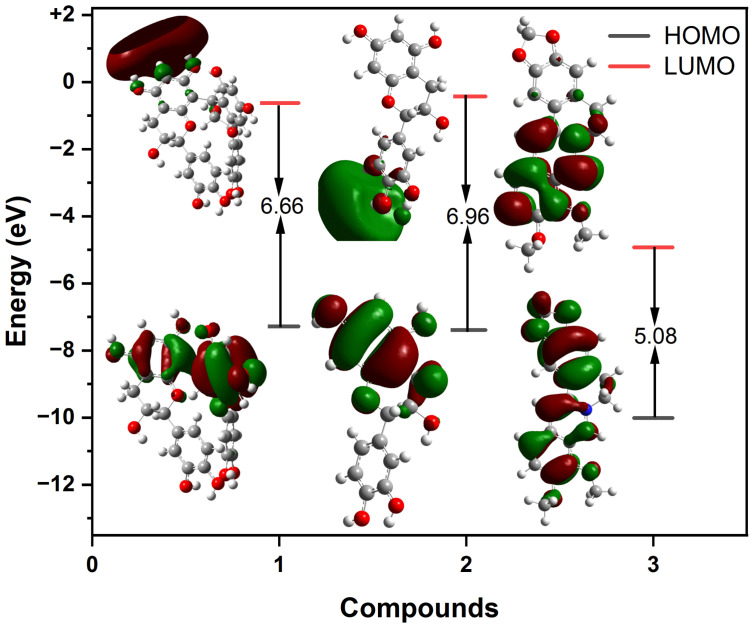
HOMO-LUMO energy diagrams and interfacial plots of (**1**) Compound **1**, (**2**) Compound **2**, and (**3**) Compound **3** (berberine chloride). The red and green isosurfaces represent the negative and positive phases of the molecular orbital wavefunction, respectively.

The frontier molecular orbital (FMO) analysis, particularly the energy gap (ΔEgap) between the highest occupied molecular orbital (HOMO) and the lowest unoccupied molecular orbital (LUMO), is a fundamental approach for predicting the kinetic stability and chemical reactivity of bioactive compounds [39]. According to FMO theory, a molecule with a larger energy gap is considered “hard”, exhibiting higher kinetic stability and lower polarizability. Conversely, a smaller energy gap indicates a “soft” molecule, which is typically more reactive and more prone to electronic excitation [40]. The HOMO–LUMO distributions and energy levels of the purified compounds (**1** and **2**) and berberine chloride (**3**) are visualized in Figure 4, with specific energy values summarized in Table 4.

As observed in Table 4, the reference compound **3** exhibits the narrowest energy gap ΔE_gap_ = 5.09 eV), whereas the purified compounds **1** and **2** display significantly larger gaps of 6.66 eV and 6.97 eV, respectively. This substantial increase in the energy gap suggests that compounds **1** and **2** possess higher kinetic stability compared to the reference. To quantitatively assess the chemical behavior, global reactivity descriptors including ionization potential (*I*), electron affinity (*A*), chemical hardness (η), softness (*S*), electronegativity (χ), and electrophilicity index (ω) were calculated based on Koopmans’ theorem [41] and standard DFT-based equations [42,43]. The results are presented in Table 5.

The data in Table 5 further corroborates these findings. Chemical hardness (η) follows the order **2** (3.48 eV) > **1** (3.33 eV) > **3** (2.54 eV). Since hardness is directly related to the resistance against electron cloud deformation, the higher η values of **1** and **2** indicate that they are chemically harder and less prone to spontaneous decomposition or non-specific reactions [44,45]. Conversely, compound **3** shows the highest softness (*S* = 0.20 eV^−1^), aligning with its higher reactivity but also implying a potential for lower selectivity.

Notably, the electrophilicity index (ω), which reflects the propensity of a molecule to accept electrons, shows a marked difference among the compounds. The reference compound **3** exhibits a relatively high ω value (10.98 eV), indicating a stronger electrophilic character, whereas the purified compounds **1** (ω = 2.34 eV) and **2** (ω = 2.19 eV) display lower electrophilicity. This variation may influence their chemical reactivity and interactions with biological targets.

#### 2.4.3. Molecular Dynamics Simulation and Complex Stability

**Figure 5:**
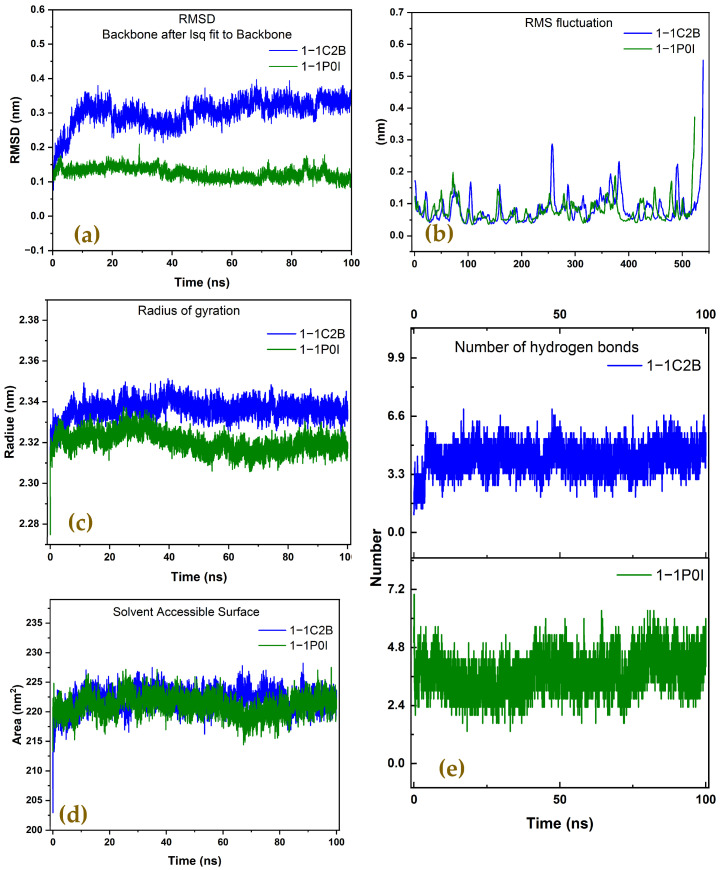
RMSD vs. time plot (**a**), RMS function plot of amino acid residues (**b**), Time-dependent Radius of Gyration (Rg) plot (**c**), and Time-dependent Solvent Accessible Surface Area (SASA) plot (**d**) for the complexes of compound 1 with 1C2B and 1P0I and Time-dependent number of hydrogen bonds plot for the 1–1C2B and 1–1P0I complexes (**e**).

**Figure 6:**
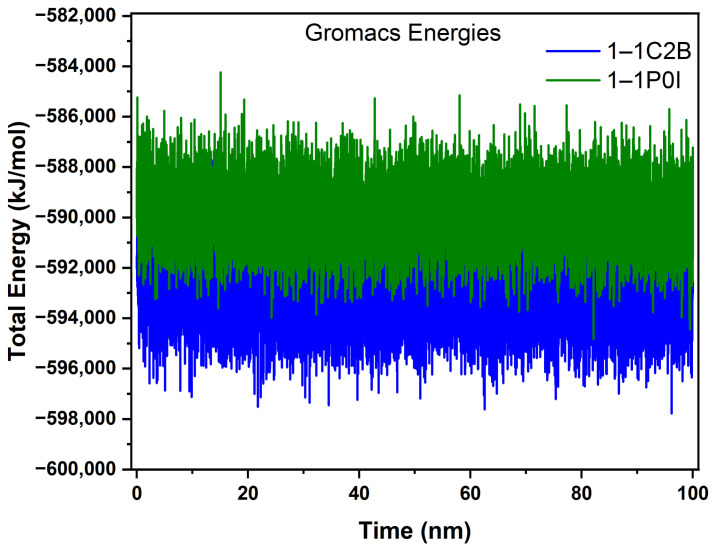
Time-dependent Total Energy plot for the 1–1C2B and 1–1P0I complexes.

To validate the stability of the docking predictions and elucidate the dynamic behavior of Compound **1** within the enzyme pockets, 100 ns molecular dynamics (MD) simulations were performed. The structural stability and conformational evolution of the 1–1C2B (AChE) and 1–1P0I (BChE) complexes were assessed using RMSD, RMSF, radius of gyration (Rg), solvent-accessible surface area (SASA), and hydrogen-bond analyses. The molecular dynamics (MD) simulations were performed in triplicate. The averaged results are shown in Figure 5 and Figure 6, while the results from individual runs are provided in the Supplementary Information (Figures S7–S10).

The root mean square deviation (RMSD) of the protein backbone (Figure 5a) was used as a primary indicator of system equilibration. The 1–1C2B complex (blue) exhibited initial fluctuations up to 0.31 nm during the equilibration phase (0–10 ns), followed by stabilization with RMSD values in the range of 0.21–0.40 nm. All subsequent parameters were calculated based on the equilibrated trajectory after 10 ns. This behavior suggests an induced-fit mechanism, in which conformational adjustments of the enzyme accommodate ligand binding [46]. In contrast, the 1–1P0I complex (green) showed greater rigidity, maintaining a stable plateau between 0.07 and 0.21 nm throughout the simulation. Both systems remained below the commonly accepted stability threshold of 0.3 nm for protein–ligand complexes [47]. Local flexibility was evaluated using root mean square fluctuation (RMSF) analysis (Figure 5b). In both complexes, active-site residues displayed minimal fluctuations (<0.29 nm), indicating strong ligand engagement that restricts residue mobility. Higher fluctuation peaks were confined to loop regions (e.g., residues 70–80 and 280–290 in AChE), which are intrinsically flexible and located away from the binding pocket. This profile is consistent with stable inhibition, where the ligand acts as a “molecular glue” that stabilizes the catalytic domain [48].

To further assess structural integrity and compactness, Rg and SASA were analyzed simultaneously (Figure 5c,d). Both parameters remained stable, showing no significant increasing trends throughout the simulation. The Rg values averaged approximately 2.34 nm for 1–1C2B and 2.32 nm for 1–1P0I, confirming maintenance of compact tertiary structures without unfolding or expansion [49]. This observation is supported by the SASA profiles, which fluctuated around ~222 nm^2^ and ~221 nm^2^ for 1–1C2B and 1–1P0I, respectively. The consistent SASA values indicate that the hydrophobic cores remained shielded from solvent exposure, suggesting that ligand binding did not disrupt overall protein folding.

Hydrogen-bond analysis (Figure 5e) provided additional insight into the stability mechanisms. The 1–1C2B complex (blue) maintained a dense interaction network, with an average of 4.29 hydrogen bonds over the 100 ns trajectory. These directional polar interactions may explain the relatively higher RMSD values, reflecting adaptive backbone motions that optimize bonding interactions. In contrast, the 1–1P0I complex (green) formed fewer hydrogen bonds (average of 3.72); however, its exceptionally low RMSD (0.07–0.21 nm) suggests that stability is primarily governed by hydrophobic packing and steric complementarity (lock-and-key fit) rather than electrostatic interactions alone [50].

Finally, thermodynamic stability was confirmed by total energy trajectories (Figure 6). The energy profiles remained stable throughout the simulation, oscillating around −593,136 kJ/mol and −589,856 kJ/mol for 1–1C2B and 1–1P0I, respectively. The absence of significant energy drift indicates that both systems reached equilibrium and that ligand–protein interactions were energetically favorable over time.

#### 2.4.4. In Silico Pharmacokinetic and Toxicity Profiling (ADMET)

Evaluation of physicochemical properties and ADMET (Absorption, Distribution, Metabolism, Excretion, and Toxicity) parameters is essential for predicting drug-likeness and minimizing late-stage failure in drug discovery [51]. Compounds **1** and **2**, together with the reference berberine chloride (**3**), were assessed using SwissADME [19] and pkCSM [51].

Lipinski’s Rule of Five (Ro5) analysis (Table 6) shows that the reference compound (**3**) fully satisfies Ro5 criteria, while compound **2** exhibits acceptable drug-likeness with only one violation and favorable lipophilicity (LogP = 1.55). In contrast, compound **1** presents four violations, mainly due to its higher molecular weight (578 Da) and elevated TPSA (220.76 Å^2^), which may reduce passive oral absorption. However, such deviations are often associated with structurally complex high-affinity ligands, and formulation strategies may help mitigate pharmacokinetic limitations [52,53].

ADMET predictions (Table 7) indicate clear differences in safety profiles. Compound **1** demonstrates the most favorable safety characteristics, being predicted as non-mutagenic and non-hepatotoxic, whereas compound **2** shows potential mutagenicity and the reference compound is predicted to exhibit both mutagenic and hepatotoxic risks. Considering that toxicity is a major cause of drug attrition, this profile highlights compound **1** as a safer candidate [54].

In terms of metabolism, compound **1** does not inhibit key CYP enzymes, including CYP3A4, suggesting lower risk of drug–drug interactions compared with the reference compound [39]. Moreover, its low BBB permeability (logBB = −2.09) implies limited CNS exposure and potentially reduced neurological side effects.

Despite deviations from Ro5 that may influence oral absorption, the superior safety profile and metabolic stability of compound **1** identify it as the most promising lead scaffold. Future optimization may focus on nanocarrier-based formulations to enhance bioavailability while maintaining its safety advantages [40].

## 3. Materials and Methods

### 3.1. In Silico Evaluation of Cholinesterase

*Terminalia triptera* Stapf. was collected from Yok Don National Park, Dak Lak Province, Vietnam. The species was identified by Thi Huong Tran, a botanist at Tay Nguyen University. A voucher specimen (Code/Registration No.: Nguyen04052O24.IBE.TNU) has been deposited at the Institute of Biotechnology and Environment, Tay Nguyen University, Dak Lak 630000, Vietnam. After collection, the plant material was air-dried, stored in polyethylene (PE) bags, and maintained at −30 °C prior to further analysis. Acetylcholinesterase (AChE), butyrylcholinesterase (BuChE), and berberine chloride were obtained from Sigma-Aldrich (St. Louis, MO, USA). All solvents, reagents, and supplementary chemicals used throughout the experiments were of analytical grade to ensure reliability and reproducibility of the results. 

### 3.2. Cholinesterase Inhibitory Assay

Cholinesterase inhibition (AChE and BChE) was evaluated using a modified Ellman’s colorimetric method [55]. The reaction mixture consisted of enzyme solution (40 µL), phosphate buffer (80 µL), and test sample or reference inhibitor (berberine chloride, 40 µL) at different concentrations, followed by pre-incubation at room temperature for 15 min. Subsequently, substrate solution (40 µL) and DTNB reagent (5,5′-dithiobis(2-nitrobenzoic acid), 0.005 M in distilled water, 40 µL) were added, and the reaction was further incubated for 15 min. Absorbance was measured at 412 nm using an iMark™ Microplate Absorbance Reader (Bio-Rad Laboratories, Hercules, CA, USA).

Blank controls were prepared under identical conditions, replacing the sample with buffer. Acetylcholinesterase (0.77 U/mL, electric eel) with acetylthiocholine iodide (0.003 M) was prepared in 0.05 M phosphate buffer (pH 8.0), while butyrylcholinesterase (0.13 U/mL, equine serum) with butyrylthiocholine iodide (0.003 M) was prepared in 0.05 M phosphate buffer (pH 7.5). PG was dissolved in dimethyl sulfoxide, and berberine chloride was prepared in methanol. The inhibition percentage was calculated as [(Abs_control − Abs_sample)/Abs_control] × 100. IC_50_ values were determined from dose–response curves, where lower IC_50_ values indicate higher inhibitory potency.

### 3.3. Preparation of Medicinal Plant Extract, Extraction, Purification of Active Compounds

Bioassay-guided fractionation based on cholinesterase inhibitory activity was employed to isolate active constituents from the methanolic trunk bark extract of *Terminalia triptera*. The crude extract (TT, 27.75 g) was subjected to Diaion HP-20 column chromatography and eluted with a stepwise MeOH/H_2_O gradient (15%, 30%, 45%, 60%, and 100% MeOH), affording five fractions: TT-1 (10.05 g), TT-2 (3.95 g), TT-3 (8.1 g), TT-4 (3.85 g), and TT-5 (1.45 g). Among these, fraction TT-4 showed the strongest inhibitory activity against acetylcholinesterase (AChE) and butyrylcholinesterase (BChE) and was therefore selected for further purification. Fraction TT-4 was subsequently separated using Sephadex LH-20 column chromatography with MeOH/H_2_O to yield three sub-fractions (TT-4.1–TT-4.3). The most active sub-fraction, TT-4.2 (0.45 g), was further purified by preparative HPLC (35% MeOH), resulting in the isolation of two pure compounds, Compd **1** (62.85 mg) and Compd **2** (3.3 mg). These compounds’ chemical structures were identified using NMR, HESI-MS, HPLC analysis and comparison with previous reports.

### 3.4. Computation

Molecular Docking: The crystal structures of AChE (PDB ID: 1C2B) and BChE (PDB ID: 1P0I) were retrieved from the RCSB Protein Data Bank. The docking process was executed using AutoDock Vina version 1.2.5 [36] integrated within UCSF Chimera 1.18 [56]. For each protein, a specific grid box was defined to cover the active site residues as follows: For AChE (1C2B) The grid center was set at (x = 33.2163, y = 84.3, z = 28.2875) with a size of (x = 29.957, y = 30, z = 31.7651) Å [57]. For BChE (1P0I) The grid center was set at (x = 131.906, y = 113.09, z = 40.3581) with a size of (x = 40, y = 24.397, z = 31.7617) Å [58]. The best-scored binding poses were selected for further analysis. The 2D ligand-protein interaction diagrams were generated using LigPlot+ v2.3 [59] to visualize hydrogen bonding and hydrophobic contacts.

DFT Calculations: The ground-state geometries of all compounds were optimized using Gaussian 09 [60] with Density Functional Theory (DFT) at the M06-2X level [61] and the 6-311++G(d,p) basis set. The M06-2X functional was chosen for its reliable description of non-covalent interactions and thermochemical properties. Harmonic frequency analyses at the same level confirmed true minima (no imaginary frequencies) and provided thermodynamic parameters.

Molecular Dynamics Simulations: Molecular dynamics (MD) simulations were performed using GROMACS 2025.02 [62] to evaluate ligand–protein stability. The CHARMM36 force field [63] was applied for protein topology, while ligand parameters were generated compatible with CHARMM36 (e.g., via CGenFF). Systems were solvated in a cubic TIP3P water box and neutralized with 0.15 M NaCl. After energy minimization (steepest descent), equilibration was conducted under NVT and NPT ensembles (100 ps each). Production simulations ran for 100 ns at 300 K and 1 bar. Trajectories were analyzed using RMSD and RMSF via GROMACS tools.

ADMET and Toxicity Prediction: The physicochemical and pharmacokinetic properties (Absorption, Distribution, Metabolism, and Excretion; ADME) were predicted using the SwissADME web server [19]. Drug-likeness was evaluated according to Lipinski’s Rule of Five, while toxicity profiles were assessed using the pkCSM database [51].

## 4. Conclusions

This study provides the first comprehensive evidence of the cholinesterase inhibitory potential of *Terminalia triptera* Stapf., emphasizing its value as a source of bioactive metabolites for Alzheimer’s disease research. Bioassay-guided isolation led to the identification of two flavan-3-ol derivatives, epicatechin-(4β→8)-*ent*-catechin (**1**) and (−)-catechin (**2**), reported for the first time from this species. The dimeric compound 1 demonstrated notable dual inhibition against AChE and BChE, surpassing berberine chloride. DFT analysis indicated high kinetic stability and low electrophilicity, suggesting a favorable safety profile. Molecular docking and 100 ns molecular dynamics simulations further confirmed stable binding within enzyme catalytic sites. Overall, these results highlight *T. triptera* dimeric proanthocyanidins as promising multi-target anti-Alzheimer candidates, supporting further pharmacological evaluation, including in vivo studies and clinical investigations.

## Figures and Tables

**Table 1:** Cholinesterase Inhibitory effect of *Terminalia triptera* Stapf. and other reported medicinal plants collected in the Central Higland of Vietnam.

Herbal Species	Parts Used	Cholinesterase Inhibitory Effect IC_50_ (µg/mL)		Ref.
		AChE	BChE	
*Terminalia triptera* Stapf.	Trunk bark	335.42 ± 8.62	405.35 ± 9.03	This study
Berberine chloride	Commercial Compd.	348.28 ± 5.78	368.35 ± 6.43	
*Tetrastigma erubescens*	Aerial parts extract	730.6	ND	Phan et al. (2025) [20]
*Eclipta prostrata* L.	Aerial parts extract	76.8 ± 0.8	ND	Phan et al. (2023) [21]
*Dillenia ovata*	Leaves extract	110 ± 10	ND	Doan et al. (2023) [22]
*Dillenia ovata*	Trunk bark extract	710 ± 10	ND	Doan et al. (2023) [22]
*Dillenia ovata*	Branch extract	400 ± 10	ND	Doan et al. (2023) [22]
*Sophora flavescens*	Root extract	107.7 ± 2.3	ND	Thang et al. (2022) [23]

ND = Not determined, IC_50_ values expressed as mean ± SD when available, standard reference inhibitor: berberine chloride.

**Table 2:** Cholinesterase inhibition of purified compounds and berberine chloride.

Sample/Compound	Cholinesterase Inhibition (mol/L)	
	AChE	BChE
Epicatechin-(4β→8)-*ent*-catechin (**1**)	4.41 × 10^−4^	4.75 × 10^−4^
(−)-Catechin (**2**)	11.9 × 10^−4^	12.1 × 10^−4^
Berberine chloride (Positive control)	8.97 × 10^−4^	9.32 × 10^−4^

All experiments were performed in triplicate (*n* = 3). Data are presented as mean ± SD. AChE: acetylcholinesterase; BChE: butyrylcholinesterase.

**Table 3:** Binding affinities and interacting residues of the purified compounds with AChE and BChE.

Compound	AChE (1C2B)		BChE (1P0I)	
	Binding Energy (kcal/mol)	Key Interacting Residues	Binding Energy (kcal/mol)	Key Interacting Residues
Epicatechin-(4β→8)-*ent*-catechin (**1**)	−9.8	Leu72, Thr71, Tyr68, Tyr120, Ser289, Glu288	−11.9	Trp79, Leu283, Ser284, Gly113, Ser195
(−)-Catechin (**2**)	−8.3	Tyr120, Trp282, Ser289, Arg292	−9.1	His433, Tyr329, Asp67, Asn65
Berberine chloride (Positive control)	−8.5	Phe291	−8.7	Tyr435, Met432, Trp425, Pro282, Thr281, Ser284, Gly113, Gln116, Gly114

**Table 4:** Frontier molecular orbital energies and energy gap values of the studied compounds.

Compound	E_HOMO_ (eV)	E_LUMO_ (eV)	ΔE_gap_ (eV)
**1**	−7.28	−0.62	6.66
**2**	−7.39	−0.43	6.97
**3**	−10.01	−4.93	5.09

Epicatechin-(4β→8)-ent-catechin (**1**), (−)-catechin (**2**), and berberine chloride (**3**, positive control). EHOMO and ELUMO correspond to the highest occupied molecular orbital and lowest unoccupied molecular orbital energies (eV), respectively; ΔEgap = ELUMO − EHOMO.

**Table 5:** Global chemical reactivity descriptors of the studied compounds.

Compound	*I* (eV)	*A* (eV)	χ (eV)	η (eV)	*S* (eV^−1^)	ω (eV)
**1**	7.28	0.62	3.95	3.33	0.15	2.34
**2**	7.39	0.43	3.91	3.48	0.14	2.19
**3**	10.01	4.93	7.47	2.54	0.20	10.98

Epicatechin-(4β→8)-*ent*-catechin (**1**), (−)-catechin (**2**), and berberine chloride (**3**, positive control). *I*: ionization potential; *A*: electron affinity; χ: electronegativity; η: chemical hardness; *S*: chemical softness; ω: electrophilicity index. All values are expressed in eV (except *S* in eV^−1^).

**Table 6:** Physicochemical properties and drug-likeness analysis based on Lipinski’s Rule of Five.

Parameter	1	2	3	Ideal Limit [49]
MW (g/mol)	578.00	290.00	337.0	<500 Da
H-bond Donors	10	5	0	<5
H-bond Acceptors	12	6	4	<10
LogP	3.00	1.55	2.73	<5
Molar Refractivity	143.39	72.62	93.03	Between 40–130
Rotatable Bonds	3	1	2	<10
TPSA (Å^2^)	220.76	110.38	40.80	<90
Lipinski Violations	4	1	0	0

**Table 7:** In silico ADMET (Absorption, Distribution, Metabolism, Excretion, and Toxicity) profiling.

Category	Parameter	1	2	3	Unit
Absorption	Intestinal Absorption	49.768	72.619	79.298	% Absorbed
Distribution	BBB Permeability	−2.092	−1.069	0.241	log BB
	CNS Permeability	−4.231	−3.403	−1.529	log PS
Metabolism	CYP3A4 Inhibitor	No	No	Yes	Yes/No
	CYP2D6 Inhibitor	No	No	No	Yes/No
	CYP1A2 Inhibitor	No	No	No	Yes/No
Excretion	Total Clearance	0.228	0.29	1.618	log mL/min/kg
	Renal OCT2 Substrate	No	No	No	Yes/No
Toxicity	AMES Toxicity	No	Yes	Yes	Yes/No
	Hepatotoxicity	No	No	Yes	Yes/No
	hERG II Inhibitor	No	No	No	Yes/No

## Data Availability

All relevant data are contained within the paper. The data analyzed in this study are available from the corresponding authors upon reasonable request.

## Supplementary Materials

The following supporting information can be downloaded at: https://www.mdpi.com/article/10.3390/molecules31071113/s1, Figure S1: High-resolution ESI-MS spectrum of compound **1** (epicatechin-(4β→8)-*ent*-catechin) recorded in negative ion mode; Figure S2: ^1^H NMR (600 MHz, pyridine-d5) spectrum of compound **1** (epicatechin-(4β→8)-*ent*-catechin); Figure S3: 13C NMR (150 MHz, MeOH- d4) spectrum of compound **1** (epicatechin-(4β→8)-*ent*-catechin); Figure S4: High-resolution ESI-MS spectrum of compound **2** [(−)-catechin] recorded in negative ion mode; Figure S5: ^1^H NMR (500 MHz, MeOH-d4) spectrum of compound **2** [(−)-catechin]; Figure S6: 13C NMR (125 MHz, MeOH- d4) spectrum of compound **2** [(−)-catechin]; Figure S7: RMSD vs. time (a), RMSF of amino acid residues (b), and time-dependent radius of gyration (Rg) (c) for the complexes of compound **1** with 1C2B from three independent MD simulations (replicates); Figure S8: Solvent-accessible surface area (SASA) (a), number of hydrogen bonds (b), and total energy (c) for the complexes of compound **1** with 1C2B from three independent MD simulations (replicates); Figure S9: RMSD vs. time (a), RMSF of amino acid residues (b), and time-dependent radius of gyration (Rg) (c) for the complexes of compound **1** with 1-1P0I from three independent MD simulations (replicates); Figure S10: Solvent-accessible surface area (SASA) (a), number of hydrogen bonds (b), and total energy (c) for the complexes of compound **1** with 1-1P0I from three independent MD simulations (replicates); Table S1: Bio-guided Iolation of Cholinesterase Inhibitors from *Terminalia triptera* Stapf.

## Abbreviations

The following abbreviations are used in this manuscript:

AD	Alzheimer’s Disease
AChE	Acetylcholinesterase
BChE/BuChE	Butyrylcholinesterase
ChEIs	Cholinesterase Inhibitors
Aβ	Amyloid-beta
DFT	Density Functional Theory
HOMO	Highest Occupied Molecular Orbital
LUMO	Lowest Unoccupied Molecular Orbital
MD	Molecular Dynamics
ADL	Activities of Daily Living
DALYs	Disability-Adjusted Life Years
CAS	Catalytic Anionic Site
PAS	Peripheral Anionic Site
IC_50_	Half-maximal Inhibitory Concentration
NMR	Nuclear Magnetic Resonance
MS	Mass Spectrometry
HESI-MS	Heated Electrospray Ionization Mass Spectrometry
HPLC	High-Performance Liquid Chromatography
PDB	Protein Data Bank
RMSD	Root Mean Square Deviation
RMSF	Root Mean Square Fluctuation
Rg	Radius of Gyration
SASA	Solvent-Accessible Surface Area
FMO	Frontier Molecular Orbitals
TTS	Terminalia Triptera Stem Bark Extract
TT	Terminalia Triptera Extract
