# Supplementary material for: Isolation, Identification and In Silico Evaluation of Novel Cholinesterase Inhibitors from Terminalia triptera Stapf
Source: Molecules. 2026 Mar 27;31(7):1113. doi: 10.3390/molecules31071113 (PMC13075089; doi:10.3390/molecules31071113)
Supplement: Supplementary file 1 [file molecules-31-01113-s001.zip › molecules-4188670-supplementary.pdf]

Supplementary materials:

Table S1. Bio-guided Iolation of Cholinesterase Inhibitors from *Terminalia triptera* Stapf.

Step of Purification	Samples	Tested Concentration	Cholinesterase Inhibition (%)	
			AChE	BChE
**Crude extract**	TT	500 µg/mL	81±3.1	78±1.6
**Separation via Diaon HP-20 column**	**TT-1**	500 µg/mL	61±2.2	54±0.2
	**TT-2**	500 µg/mL	54±0.7	51±1.6
	**TT-3**	500 µg/mL	70±0.9	57±0.8
	TT-4	500 µg/mL	85±1.4	81±2.2
	TT-5	500 µg/mL	53±1.4	64±2.5
**Sub-fraction via LH-20 column**	TT-4.1	500 µg/mL	71±1.7	74±0.6
	TT-4.2	500 µg/mL	97±3.4	95±1.3
	TT-4.3	500 µg/mL	80±2.8	76±2.1
**Isolation via Pre. HPLC**	Compnd 1	500 µg/mL	98±2.1	93±1.6
	Compnd 1	500 µg/mL	93±1.1	94±2.5

Note: The MeOH extract of *Terminalia triptera* (TT). The crude extract (TT) was fractionated using Diaion HP-20 column chromatography with a MeOH/H_2O gradient to yield five primary fractions: TT-1 (15% MeOH), TT-2 (30% MeOH), TT-3 (45% MeOH), TT-4 (60–100% MeOH), and TT-5 (100% MeOH). The most active fraction, TT-4, was further subjected to LH-20 column chromatography (MeOH/H_2O) to afford three sub-fractions: TT-4.1 (100% MeOH), TT-4.2 (85% MeOH), and TT-4.3 (70–85% MeOH). Sub-fraction TT-4.2 was purified by preparative HPLC (35% MeOH) to yield compound **1** [epicatechin-(4β→8)-ent-catechin] and compound **2** [(−)-catechin]

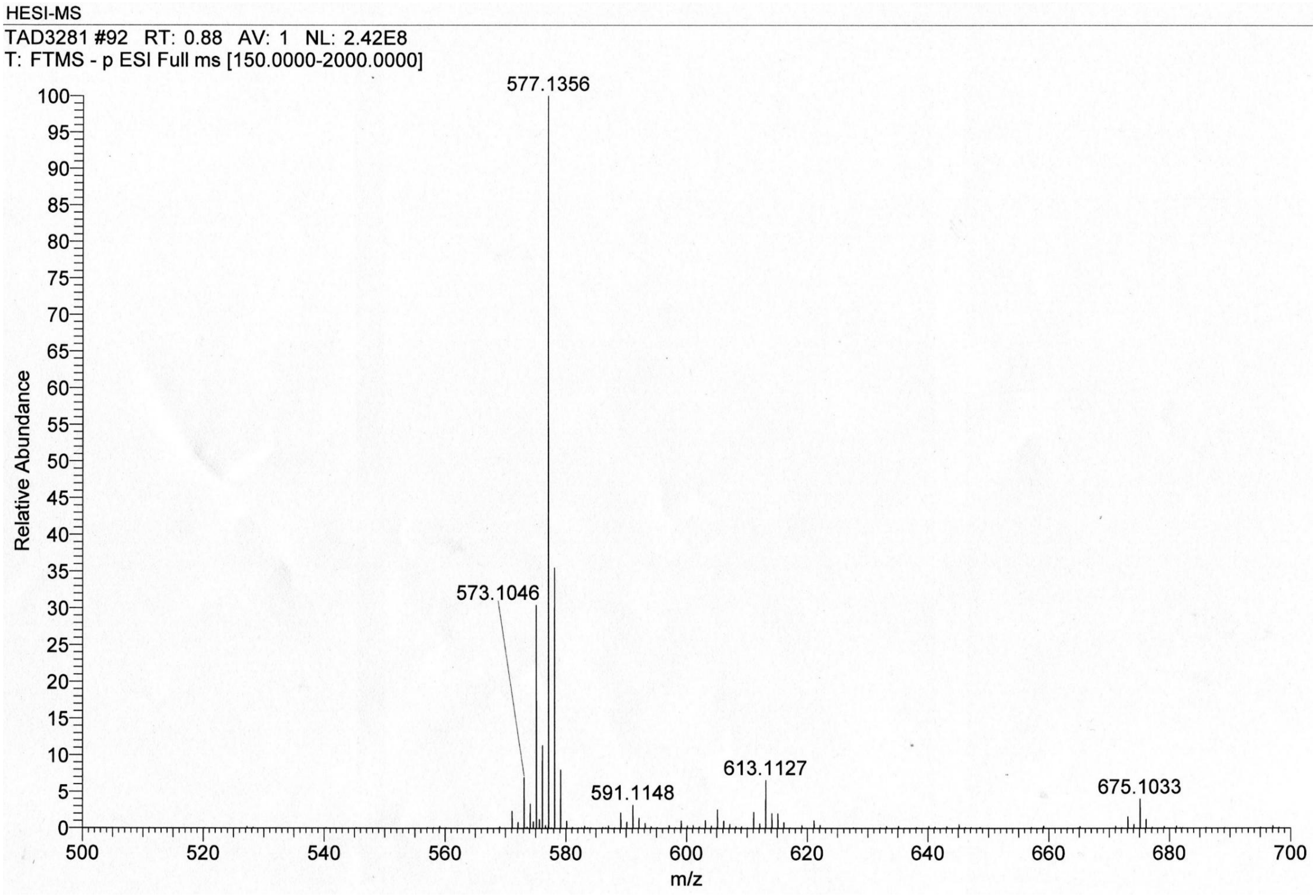

Figure S1. High-resolution ESI-MS spectrum of compound **1** (epicatechin-(4β→8)-ent-catechin) recorded in negative ion mode.

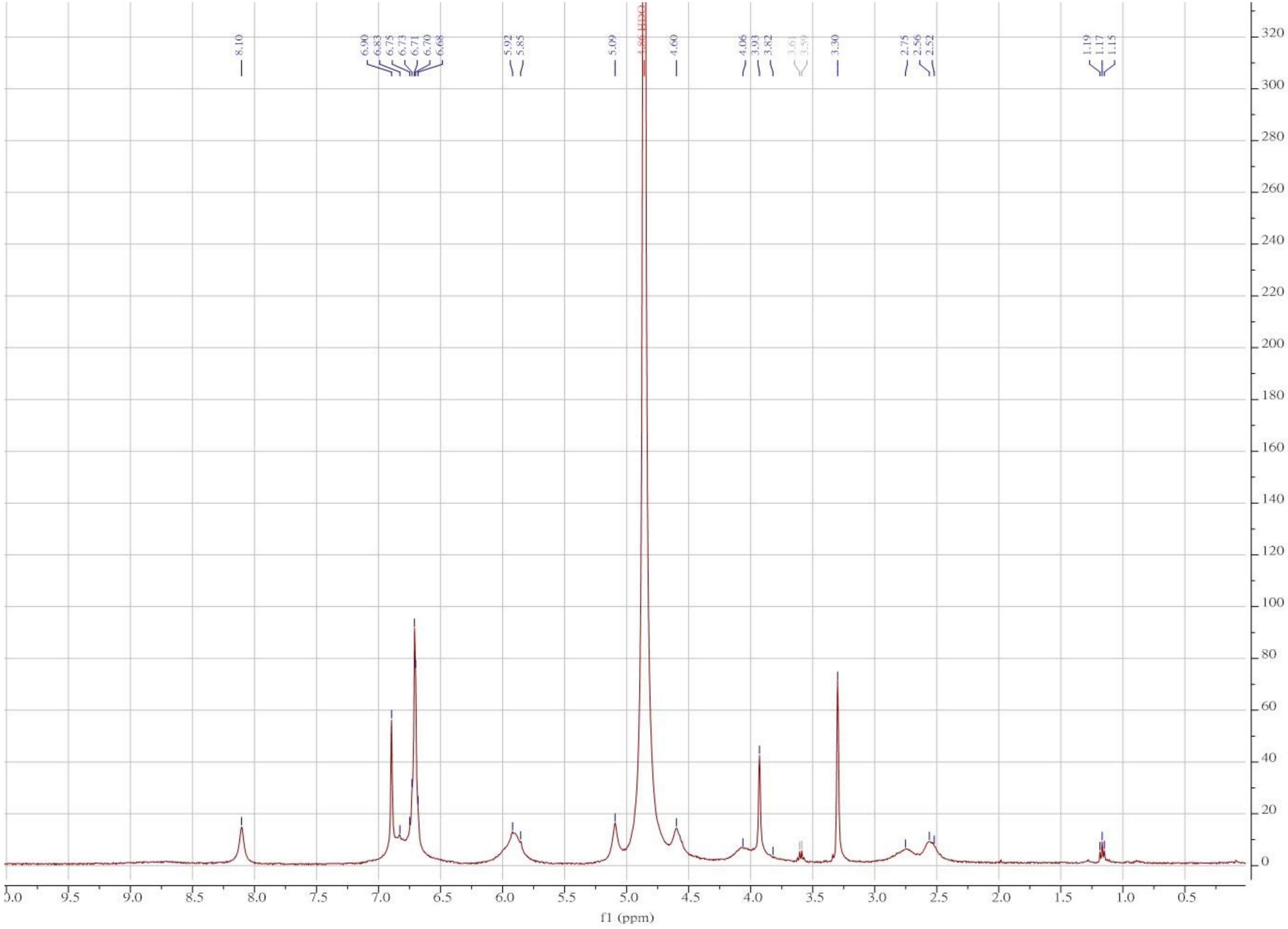

Figure S2. ^{1}H NMR (600 MHz, pyridine-*d*5) spectrum of compound 1 (epicatechin-(4β→8)-ent-catechin).

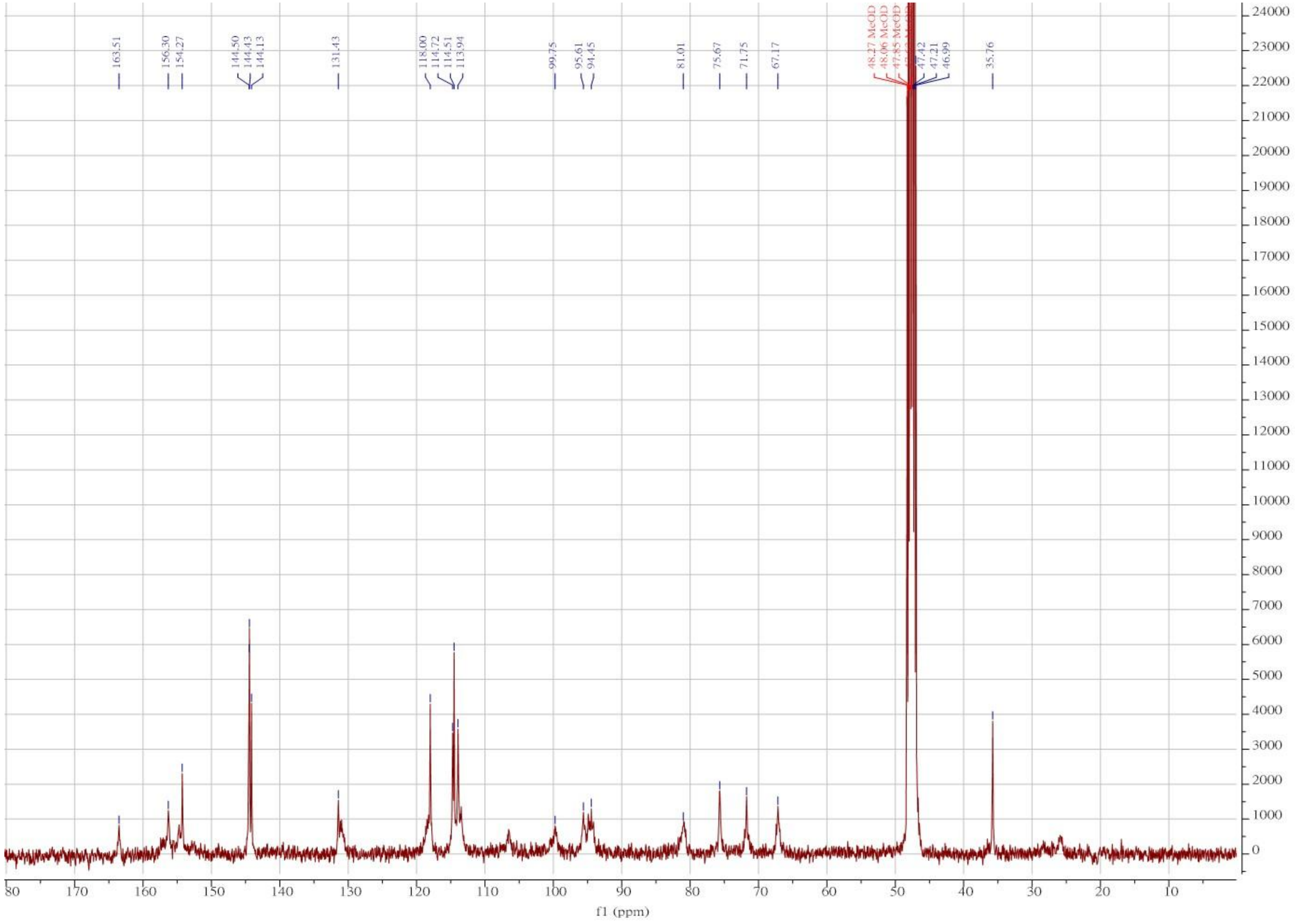

Figure S3. ^{13}C NMR (150 MHz, MeOH- *d*4) spectrum of compound **1** (epicatechin-(4β→8)-ent-catechin)

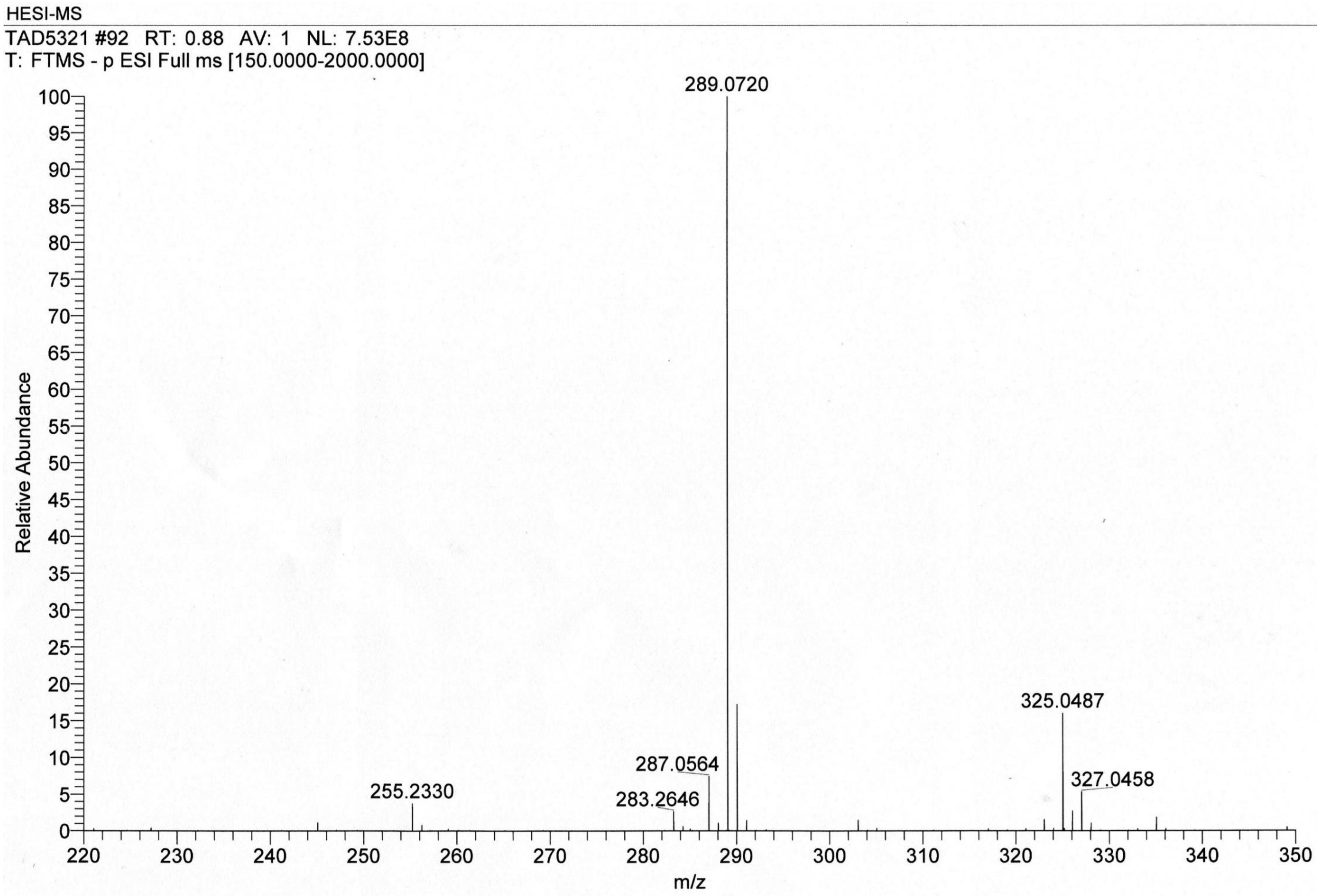

Figure S4. High-resolution ESI-MS spectrum of compound **2** [(−)-catechin] recorded in negative ion mode.

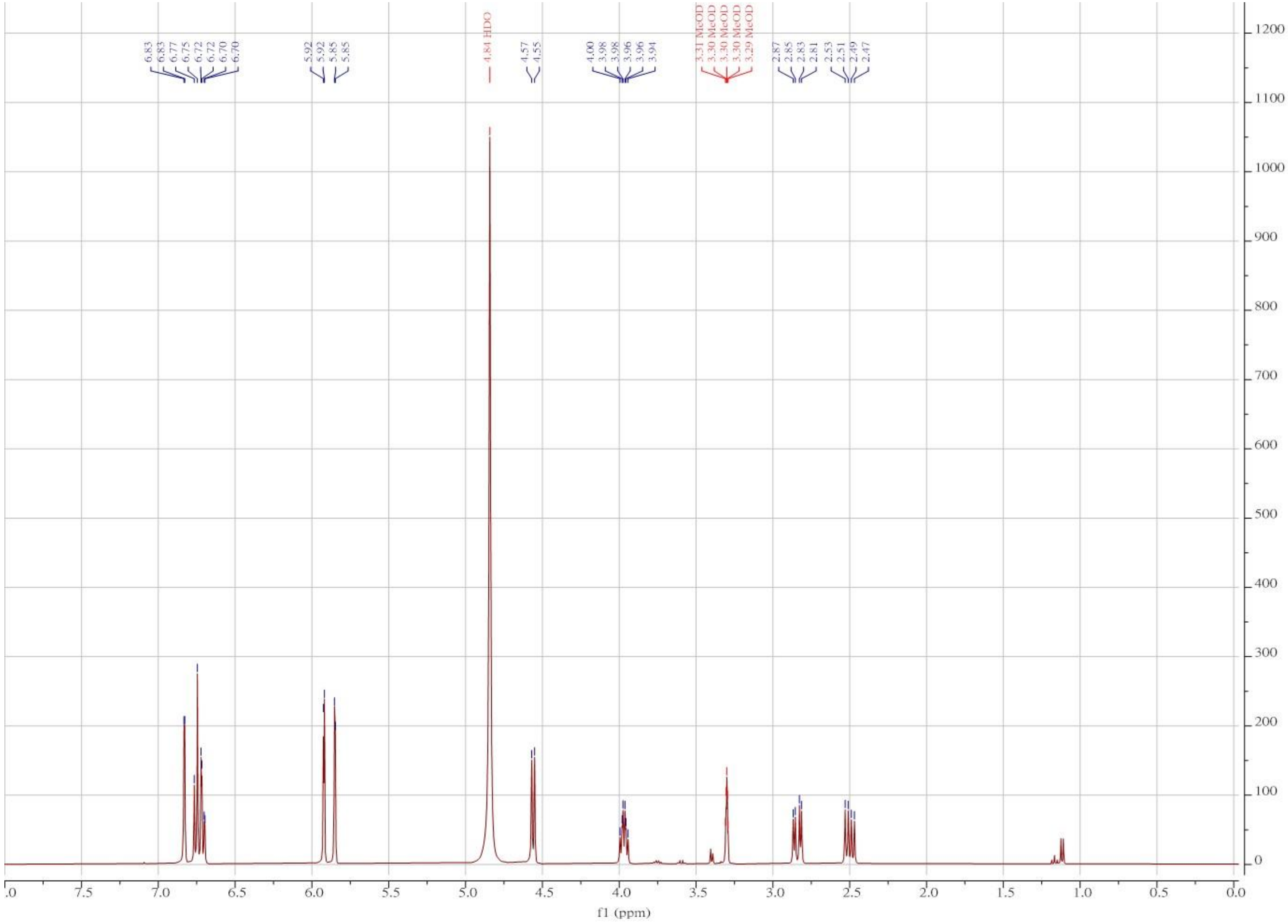

Figure S5. ^{1}H NMR (500 MHz, MeOH-*d*4) spectrum of compound **2** [(−)-catechin]

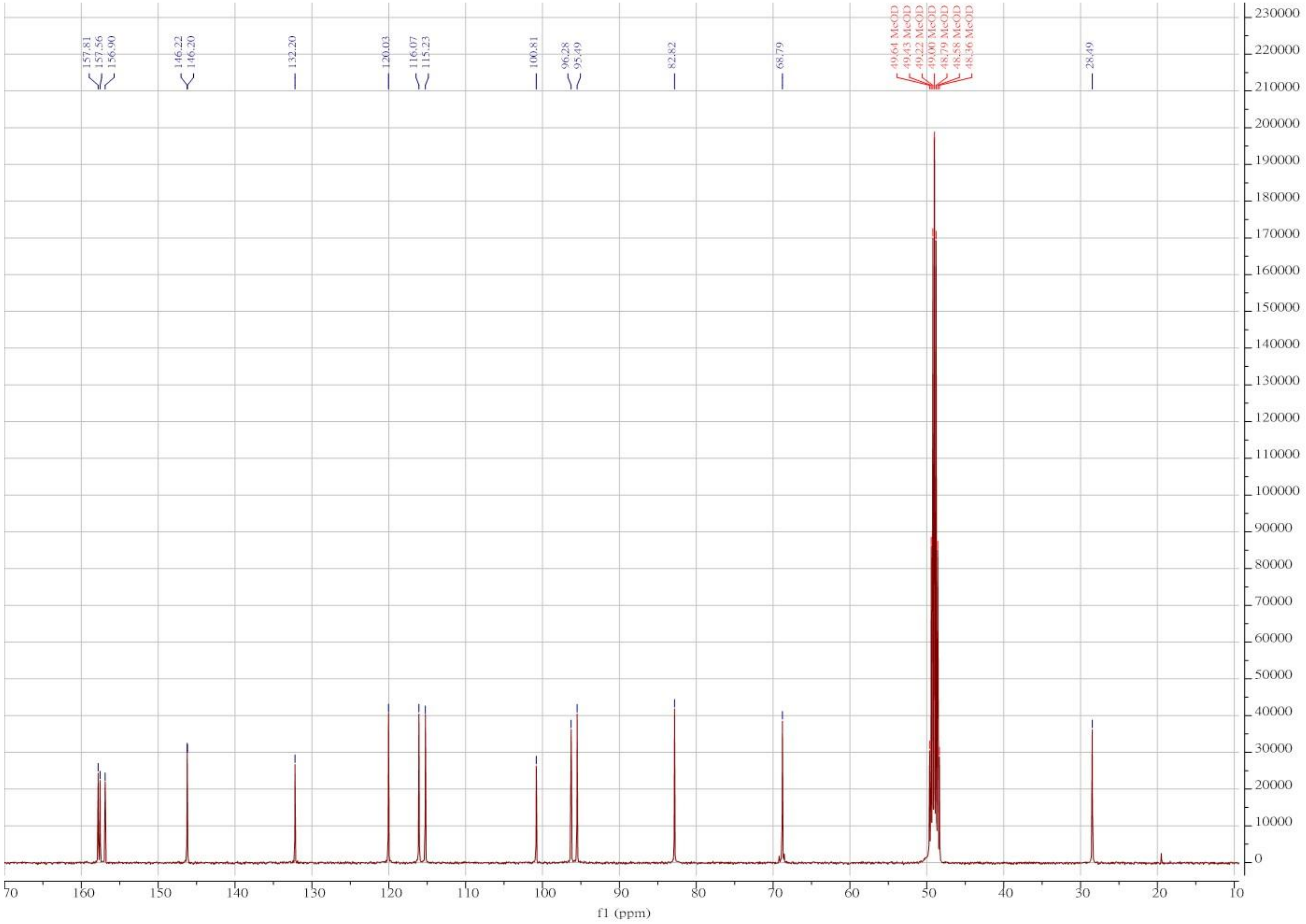

Figure S6. ^{13}C NMR (125 MHz, MeOH- *d*4) spectrum of compound **2** [(−)-catechin]

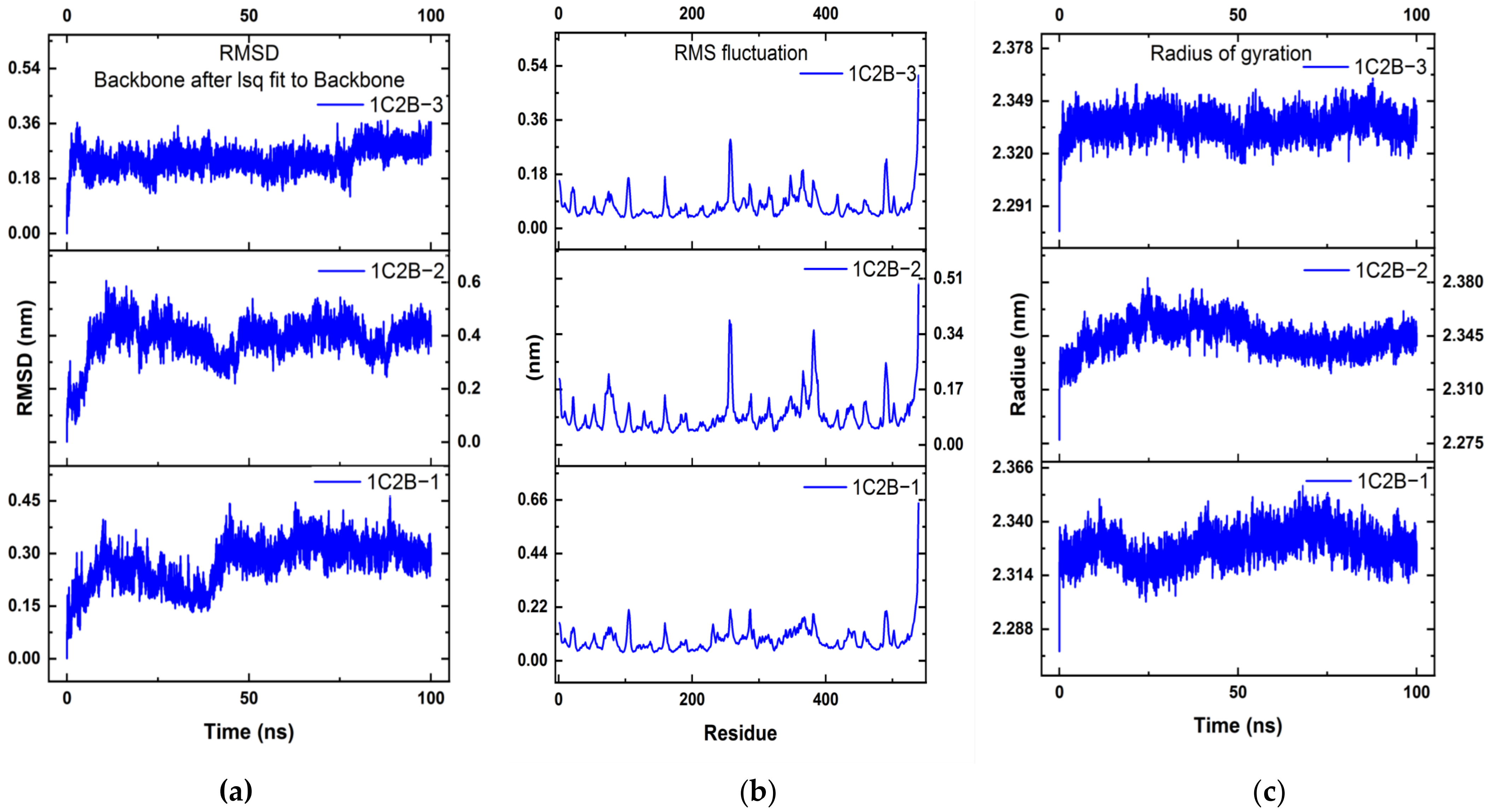

Figure S7. RMSD vs. time (a), RMSF of amino acid residues (b), and time-dependent radius of gyration (Rg) (c) for the complexes of compound **1** with 1C2B from three independent MD simulations (replicates).

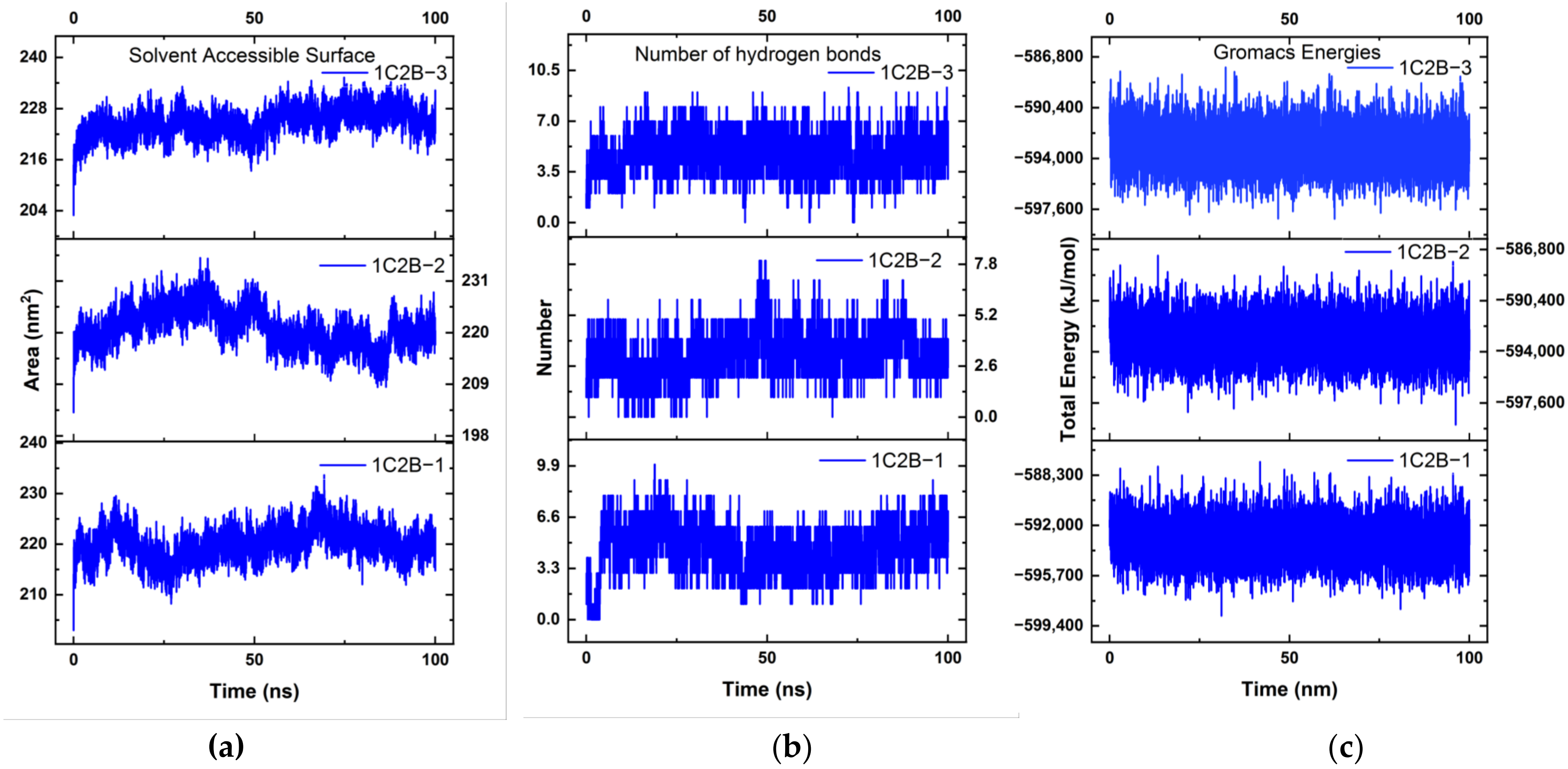

Figure S8. Solvent-accessible surface area (SASA) (a), number of hydrogen bonds (b), and total energy (c) for the complexes of compound 1 with 1C2B from three independent MD simulations (replicates).

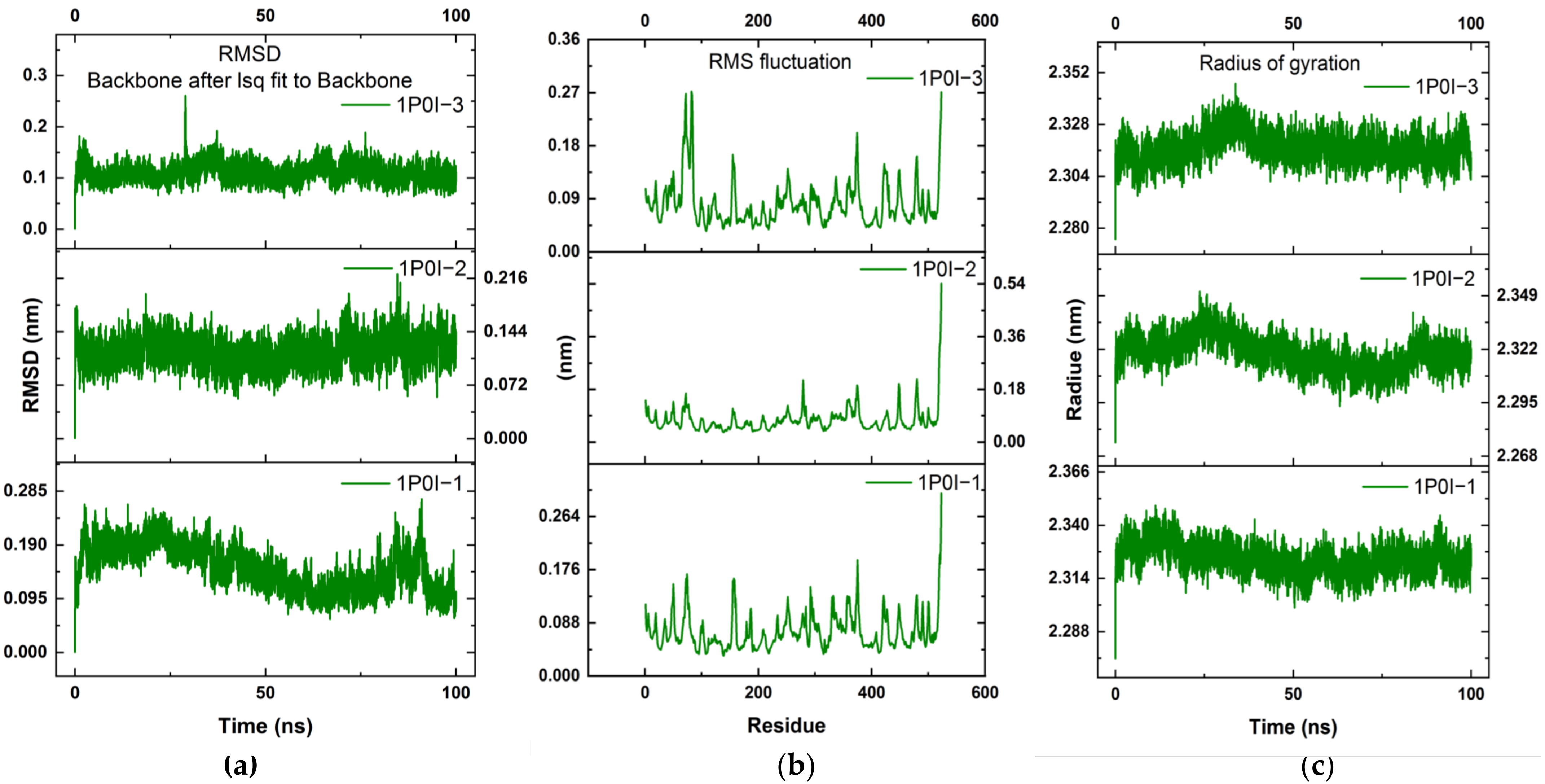

Figure S9. RMSD vs. time (a), RMSF of amino acid residues (b), and time-dependent radius of gyration (Rg) (c) for the complexes of compound **1** with 1-1P0I from three independent MD simulations (replicates).

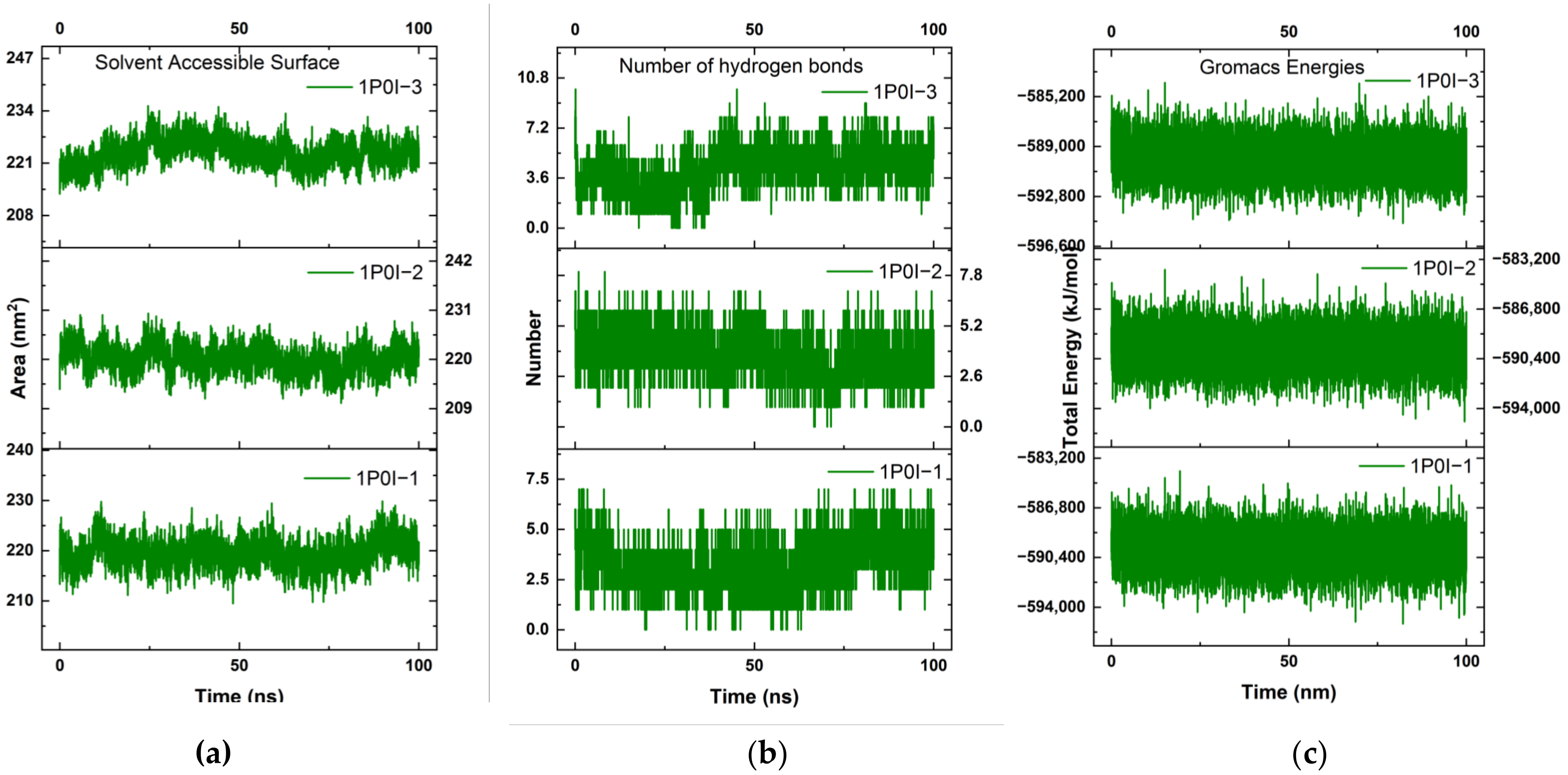

Figure S10. Solvent-accessible surface area (SASA) (a), number of hydrogen bonds (b), and total energy (c) for the complexes of compound 1 with 1-1P0I from three independent MD simulations (replicates).
